# Gender-modulated risk of coronary heart disease, diabetes and coronary mortality among Turks for three major risk factors, and residual adiposity risk

**DOI:** 10.1186/s12902-016-0134-6

**Published:** 2016-09-29

**Authors:** Günay Can, Altan Onat, Eray Yurtseven, Yusuf Karadeniz, Tuğba Akbaş-Şimşek, Ayşem Kaya, Hüsniye Yüksel

**Affiliations:** 1 Departments of Public Health, Istanbul University, Yazıcı sok. 18/5, Kocamustafapaşa, 34098 Istanbul, Turkey; 2 Departments of Cardiology, Cerrahpaşa Medical Faculty, Istanbul University, Istanbul, Turkey; 3 Department of Endocrinology and Metabolism, Ataturk University Faculty of Medicine, Erzurum, Turkey; 4 Bağcılar Educational Hospital, Istanbul, Turkey; 5 Departments of Biochemistry Laboratory, Institute of Cardiology, Istanbul University, Istanbul, Turkey

**Keywords:** Autoimmune activation, Body mass index, Coronary heart disease, Coronary mortality, Type-2 diabetes, Gender difference

## Abstract

**Background:**

We determined the proportion of the effects of body mass index (BMI) or its categories on cardiometabolic outcomes mediated through systolic blood pressure (SBP), total cholesterol and fasting glucose.

**Methods:**

Cox regression analyses were performed for incident outcomes among Turkish Adult Risk Factor study participants in whom the three mediators had been determined (*n* = 2158, age 48.5 ± 11 years). Over a mean 10.2-years’ follow-up, new coronary heart disease (CHD) developed in 406, diabetes in 284 individuals, and 149 CHD deaths occurred.

**Results:**

Hazard ratios (HR) of BMI for incident diabetes were no more than marginally attenuated by the 3 mediators including glucose, irrespective of gender. Compared to “normal-weight”, sex- and age-adjusted RRs for incident CHD of overweight and obesity were 1.40 and 2.24 (95 % CI 1.68; 2.99), respectively, in gender combined. Only three-tenths of the excess risk was retained by BMI in men, six-tenths in women. No mediation of glycemia was discerned in males, in contrast to greatest mediation in females. HR of age-adjusted continuous BMI was a significant but modest contributor to CHD mortality in each gender. While the BMI risk of CHD death was abolished by mediation of SBP in men, HR strengthened to over two-fold in women through mediation of fasting glucose.

**Conclusions:**

Mediation of adiposity by 3 traditional factors exhibited among Turkish adults strong gender dependence regarding its magnitude for CHD risk and the mediation by individual risk factors. Retention of the large part of risk for diabetes in each sex and for CHD in women likely reflects underlying autoimmune activation.

**Electronic supplementary material:**

The online version of this article (doi:10.1186/s12902-016-0134-6) contains supplementary material, which is available to authorized users.

## Background

Rise in the proportion of overweight and obese individuals represents the most prominent recent cardiovascular risk factor in Turkey and worldwide. Many studies have shown that cardiovascular risk of excess adiposity is mediated mainly by traditional risk factors such as systolic blood pressure (SBP), levels of total cholesterol and glucose, or diabetes [1–4]. The magnitude of the mediation may vary across races and by gender. In order to appropriately target the individual risk factors, the effect size of the mediating factors and the excess residual risk of adiposity are highly relevant from the public health viewpoint.

The role of BMI in cardiovascular disease in the Asia-Pacific region has been studied in a meta-analysis [5], and the association of overweight with increased coronary heart disease (CHD) risk independent of BP and cholesterol level has been investigated in another meta-analysis [6]. The most extensive quantification of the effects of high BMI, and of overweight and obesity on CHD mediated through SBP, serum cholesterol, and glucose, has been in the Global Burden of Disease (GBD) study [7], analyzing data from 97 prospective cohort studies. The HR for each 5 kg/m^2^ higher BMI was 1.27 for CHD, after adjustment for confounders. Additional adjustment for the three metabolic risk factors reduced the HRs to 1.15 indicating that 46 % of the excess risk of BMI for CHD was mediated by these factors. SBP was the most important mediator, accounting for 31 % of the excess risk on CHD. Compared with normal weight, both overweight and obesity were associated with significantly increased CHD risk, with 50 % of the excess risk of overweight and 44 % of the excess risk of obesity for CHD being mediated by the selected 3 mediators. Authors concluded that, though nearly one half of excess risk for CHD due to high BMI was mediated through 3 metabolic risk factors, maintaining optimal body weight was needed for the full benefits.

The share of Middle Eastern populations in the GBD meta-analysis comprised less than 0.5 % of the total participants and hardly 1 % of the CHD events. Of the 57161 CHD events, 88.6 % were recorded in Western cohorts. Therein elicited major findings might have limited applicability for Turkish adults, since differences may exist in how BMI affects the mediating metabolic risks in diverse populations. Moreover, sex-stratified analyses were not provided, an aspect of critical importance among Turks [8]. Thus we undertook the task of clarifying the effects of the mediating risk factors for BMI on CHD risk among Turks. Our aim was to determine, separately in the sexes, the residual risk of incident nonfatal and fatal CHD of overweight and obesity after excluding the effects of mediation of blood pressure (BP), glucose and total cholesterol levels. Finally, the “net” BMI effect was examined regarding incident diabetes as well.

## Methods

### Study sample

The TARF study is a prospective cohort study on the prevalence of cardiac disease and risk factors in adults in Turkey carried out biennially since 1990 in 59 communities scattered throughout all geographical regions of the country [9]. It comprises a random sample of the Turkish adult population, representatively stratified for sex, age, geographical regions and for rural–urban distribution. New random recruitment forming 15 % of the study sample was made in 2002/’03. Participants numbering 2287 –of whom 129 had CHD at baseline– composed the cohort of the current study. BMI, BP and fasting glucose determinations were available in the surveys encompassing the 10 years from 1997/’98 to 2007. Seventy-three % of participants had a baseline in the survey 1997/’98. The sample did not contain individuals who had been deceased prior to baseline, and 390 participants with no follow-up were excluded. The study was approved by the Ethics Committee of the Istanbul University Medical Faculty. Written informed consent for participation was obtained from all. Data were obtained by history of the past years via a questionnaire, physical examination of the cardiovascular system, sampling of blood and recording a resting 12-lead electrocardiogram.

### Measurement of risk factors

BP was measured with an aneroid sphygmomanometer (Erka, Bad Tölz, Germany) in the sitting position on the right arm, and the mean of two recordings 5 min apart was recorded. Height was measured without shoes using a measuring stick and weight without shoes in light indoor clothes using a scale. Waist circumference was measured at the level midway between the lower rib margin and the iliac crest. Cigarette smoking status was categorized into never, former and current smokers. Anyone who reported the use of alcoholic beverages once a week or more was considered as alcohol user. Physical activity was graded by the participant himself into four categories of increasing order with the aid of a scheme [9].

*Blood samples* were collected, spun at 1000 g, shipped to Istanbul and stored in deep-freeze at −75 °C, until analyzed within weeks. Serum concentrations of glucose, total cholesterol, fasting triglycerides, low-density lipoprotein (LDL)- and high-density lipoprotein (HDL)-cholesterol were determined using Cobas 500 autoanalyzer (Roche Diagnostic GmbH, Germany). Serum concentrations of apolipoprotein (apo) B and apoA-I were measured nephelometrically by BN ProSpec analyzer (Siemens Healthcare Diagnostics, Germany).

### Definitions

Participants were classified into three categories: BMI ≤25 kg/m^2^, overweight (BMI 25.0–29.9 kg/m^2^), obesity (BMI ≥30.0 kg/m^2^). *Hypertension* was defined as a blood pressure ≥140 mmHg and/or ≥90 mmHg, and/or use of antihypertensive medication. Individuals with *metabolic syndrome* (MetS) were identified when 3 out of the 5 criteria of the National Cholesterol Education Program (ATP III) [10] were met, modified for prediabetes (fasting glucose 100–125 mg/dl [11] and further for abdominal obesity using as cutpoint ≥95 cm in men, as assessed in the Turkish Adult Risk Factor study [12]. Diabetes was diagnosed with the criteria of the American Diabetes Association, namely by self-report of antidiabetic medication, or a plasma fasting glucose ≥126 mg/dl [13].

*Diagnosis of CHD* was based on the presence of angina pectoris, of a history of myocardial infarction with or without accompanying Minnesota codes of the ECG [14] or on a history of myocardial revascularization. Typical angina and, in women, age >45 years were prerequisite for a diagnosis when angina was isolated. ECG changes of “ischemic type” of greater than minor degree (Codes 1.1–2, 4.1–2, 5.1–2, 7.1) were considered as myocardial infarct sequelae or myocardial ischemia, respectively. CHD death comprised death from heart failure of coronary origin or fatal coronary event. Death was ascertained via information from first-degree relatives, records of local health personnel and/or the nation-wide Identity Participation System.

### Data analysis

Descriptive parameters were shown as mean ± standard deviation or in percentages. For several other variables with skewed distribution, values derived from log-transformed (geometric) means were used. An SD value of (for example) 1.7 indicates the factor needed to multiply or divide the mean value to obtain the limits of the SD. ANOVA analyses and pairwise comparisons with post hoc Tukey HSD were made to detect significance across more than two groups; two-sided t-tests and Pearson’s chi-square tests were used to analyze the differences between means and proportions of two groups.

Cox proportional hazard regression analyses of the BMI categories for outcomes were performed in models that adjusted basically for (sex and) age and smoking status. Participants with outcome conditions at baseline were excluded from regression analyses. The relative risk (RR) for the three adiposity categories and the hazard ratio (HR)s for the mediating factors (systolic BP, total cholesterol and fasting glucose) were estimated and expressed for each in terms of 1-SD increment. We adjusted for sex, age, smoking status, and additionally for the 3 mediators in regard to analyses involving WC. Residual adiposity risk was estimated as percentage of excess risk mediated by the 3 risk factors deducted from the excess risk of the BMI category, which we expressed also as percentage of excess risk mediated [7, 15]. A value of *p* < 0.05 on the two-tail test was considered statistically significant. Statistical analyses were performed using SPSS-10 for Windows.

## Results

The study sample consisted of 2287 participants (of whom 1199 female) at age 48.5 ± 11 years at baseline. Mean age was similar in the sexes. Of the 616 subjects in the category BMI ≤25 kg/m^2^, only 67 had BMI <20 kg/m^2^. Follow-up ranged from 2 to 16 years with a mean of 10.2 ± 4.6 years. Total follow-up amounted to 21,980 person-years. Incident CHD manifested in 406, incident diabetes in 284 persons, and CHD mortality was identified in 149 (6.9 %) individuals (yielding a CHD mortality rate of 6.56 [8.0 for men, 5.0 for women] per 1000 person-years).

**Fig. 1:**
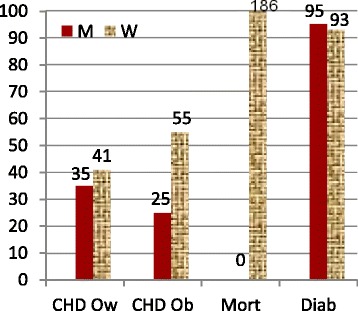
Depicts percentages of excess risks of obesity in men and women for incident coronary heart disease (CHD), type-2 diabetes and overall mortality. CHD risk is indicated separately also for overweight. It is evident that obesity in females, as distinct from males, retains substantially greater risk for CHD mortality and CHD, unmediated by the studied 3 traditional risk factors

Baseline characteristics of the sample free of CHD are presented in Table 1, stratified to BMI categories and gender. As also seen in Fig. 1, obesity existed only in 19 % of men, but in 44.4 % of women –an odds of 2.3-fold. Among the 18 variables examined, all but apoA-I and in men alcohol usage, were significantly different across the BMI categories. Though HDL-cholesterol was significantly lower in obesity than in normal-weight in each gender, apoA-I concentrations were similar. Never smokers were significantly more prevalent, current smokers less prevalent in obesity than in normal-weight, regardless of sex. Pulse pressure was higher in obese women than obese men (55.3 vs. 50.6 mmHg)

**Table 1:** Baseline characteristics of the study sample stratified to BMI categories and gender (*n* = 2158)

		BMI categories						ANOVA *p*-value
		BMI ≤25		Overweight		Obesity		
	*n*	Mean	SD	Mean	SD	Mean	SD	
Men	1015	*n* = 350		*n* = 473		*n* = 192		
Age, years	1015	47,3	12,9	48,9	11,3	50,5	11,3	,008
Phys. activity gr., I-IV	1013	2,59	1,07	2,40	1,02	2,31	1,00	,004
Waist circumf., cm	1012	83,5	7,1	96,3	6,0	106,8	8,7	<0,001
Systolic BP, mmHg	1015	117,5	17,5	128,0	19,7	139,8	24,9	<0,001
Diastolic BP, mmHg	1015	75,0	10,5	82,1	11,4	89,2	14,6	<0,001
T. cholesterol, mg/dl	1015	173,8	35,5	185,4	36,6	194,8	38,8	<0,001
HDL cholest, mg/dl	1011	41,1	13,1	36,3	10,6	35,5	10,0	<0,001
LDL-cholest, mg/dl	994	108,8	31,0	114,8	31,4	119,3	31,7	,001
Triglycerides, mg/dl	1012	121,7	70	172,0	100,6	194,6	112	<0,001
Apolipoprot.A-I, mg/dl	838	131,9	28	128,7	24,1	131	27,6	,262
Apolipoprot. B, mg/dl	824	103,8	31,2	110,4	33,8	111,5	30,9	,015
Fast. glucose, mg/dl	1015	94,7	24,3	97,9	29,8	102,8	31,2	,007
B mass index, kg/m^2^	1015	22,5	1,9	27,4	1,35	32,6	2,5	<0,001
Diabetes, yes *n*, %	1015	10	2,9	25	5,3	19	9,9	0,002
MetS, yes *n*, %	1015	41	11,7	260	55,0	142	74,0	<0,001
Hypertension, *n*, %	1015	54	15,4	171	36,2	109	56,8	<0,001
Current, former smoker, *n*	1011	216; 55	62.2; 15.9	218; 111	46.1; 23.5	81; 47	42.4; 24.6	<0,001
Alcohol usage, yes, *n*, %	1008	58	16,8	91	19,3	28	14,7	0,332
Women	1143	234		402		507		
Age, years	1143	45,0	12,5	47,6	12,1	50,4	10,6	<0,001
Phys. activity gr., I-IV	1135	2,24	,71	2,18	,67	2,08	,67	,010
Waist circumf., cm	1137	77,3	7,7	88,2	7,9	100,5	9,4	<0,001
Systolic BP, mmHg	1143	118,9	20,7	129,8	24	143,0	26,7	<0,001
Diastolic BP, mmHg	1143	75,0	11,2	82,2	12,7	88,7	14,7	<0,001
T. cholesterol, mg/dl	1143	176,9	37,4	190,7	37,2	198,2	38,7	<0,001
HDL cholest, mg/dl	1139	47,9	13,7	44,7	11,7	43,8	12, 6	<0,001
LDL-cholest, mg/dl	1115	108	32,8	120,	31,3	123,4	34,4	<0,001
Triglycerides, mg/dl	1143	110,7	80,1	131,7	78,9	155,4	88,8	<0,001
Apolipoprot.A-I, mg/dl	937	144,1	31,9	142,6	28,3	144,3	28,8	,692
Apolipoprot. B, mg/dl	944	103,7	30,7	110,4	37,3	116,3	45,0	,002
Fast. glucose, mg/dl	1143	96,3	26,7	98	23,9	102,6	30,4	,005
B mass index, kg/m^2^	1143	22,6	1,89	27,6	1,4	34,5	3,8	<0,001
Diabetes, yes *n*, %	1139	7	3,0	15	3,7	53	7	<0,001
MetS, yes *n*, %	1143	35	15,0	164	40,8	373	73,6	<0,001
Hypertension, *n*, %	1143	43	18,4	145	36,1	301	59,4	<0,001
Current, former smoker, *n*, %	1142	77; 9	32,9; 3,8	83; 23	20,6; 5.7	51; 20	10.1; 4.0	<0,001
Alcohol usage, yes, *n*, %	1138	7	3,0	2	,5	0	0	<0,001

Table 2 shows the prediction of incident CHD by BMI categories in Cox regression, by gender and adjusted by the three mediating factors in 3 models. In the model adjusted only for sex, age and smoking status, overweight disclosed an RR of 1.40 compared to normal-weight, whereas obesity had an HR 2.24 (95 % CI 1.68; 2.99) in gender combined. The addition of systolic BP, fasting glucose plus LDL- and HDL-cholesterol attenuated the HR of overweight to non-significance, but obesity retained strong significance (RR 1.56). This showed gender disparity inasmuch as while obesity in men was no longer significant, it was significant in women with an RR 1.90 (95 % CI 1.20; 3.02), retaining 61 % of the excess CHD risk independent of fasting glucose, systolic BP and total cholesterol.

**Table 2:** Cox regression models for the prediction of incident CHD by BMI categories and mediators, by gender

	Total		Men		Women	
	HR	95 % CI	HR	95 % CI	HR	95 % CI
	404/2153^a^		185/1011^a^		219/1142^a^	
Sex, female	0.93	0.73; 1.18				
Age, 11 years	**1.78**	1.64; 1.96	**1.82**	1.59; 2.08	**1.76**	1.56; 2.00
Current vs. never smoking	**1.60**	1.24; 2.07	**1.58**	1.11; 2.25	**1.65**	1.13; 2.42
Former vs. never smoking	1.28	0.91; 1.79	1.25	0.83; 1. 09	1.32	0.67; 2.58
Overweight	**1.40**	1.05; 1.85	**1.44**	1.01; 2.05	1.40	0.87; 2.24
Obesity	**2.24**	1.68; 2.99	**2.00**	1.34; 3.00	**2.48**	1.59; 3.87
Model 1						
Sex, female	*0.83*	0.68; 1.02				
Age, 11 years	**1.57**	1.43; 1.73	**1.56**	1.34; 1.78	**1.57**	1.37; 1.78
Overweight	1.18	0.89; 1.57	1.17	0.82; 1.67	1.19	0.74; 1.92
Obesity	**1.61**	1.20; 2.17	1.37	0.89; 2.10	**1.79**	1.14; 2.80
Systolic BP, 25 mmHg	**1.37**	1.24; 1.51	**1.52**	1.27; 1.82	**1.31**	1.17; 1.48
Model 2						
Sex, female	0.87	0.71; 1.07				
Age, 11 years	**1.64**	1.51; 1.80	**1.71**	1.49; 1.96	**1.61**	1.40; 1.84
Overweight	1.26	0.95; 1.67	1.31	0.92; 1.86	1.27	0.79; 2.03
Obesity	**1.87**	1.41; 2.50	**1.69**	1.12; 2.55	**2.10**	1.35; 3.25
Total cholesterol, 40 mg/dl	**1.23**	1.11; 1.37	**1.25**	1.07; 1.45	**1.23**	1.07; 1.42
Fasting glucose, 25 mg/dl	**1.12**	1.04; 1.20	1.02	0.92; 1.13	**1.20**	1.05; 1.32
Model 3	399/2098^a^		183/987^a^		216/1111^a^	
Sex, female	0.92	0.72; 1.18				
Age, 11 years	**1.62**	1.45; 1.78	**1.61**	1.39; 1.86	**1.61**	1.38; 1.84
Current vs. never smoking	**1.67**	1.28; 2.17	**1.68**	1.17; 2.42	**1.61**	1.09; 2.38
Former vs. never smoking	1.30	0.93; 1.83	1.23	0.82; 1.72	1.47	0.75; 2.90
Overweight	1.19	0.90; 1.59	1.19	0.82; 1.72	1.31	0.81; 2.11
Obesity	**1.56**	1.15; 2.12	1.30	0.83; 2.04	**1.90**	1.20; 3.02
LDL-cholesterol, 32 mg/dl	**1.14**	1.04; 1.26	*1.13*	0.98; 1.31	**1.16**	1.02; 1.32
HDL-cholesterol, 12 mg/dl	**0.89**	0.80; 0.99	0.96	0.82; 1.12	**0.83**	0.72; 0.96
Fasting glucose, 25 mg/dl	**1.12**	1.04; 1.21	1.05	0.92; 1.19	**1.20**	1.08; 1.32
Systolic BP, 25 mmHg	**1.38**	1.25; 1.52	**1.52**	1.27; 1.82	**1.32**	1.17; 1.49

Overweight and obesity prevailed in 45.6 and 18.9 % in men, in 35.2 and 44.4 % in women

Referent in adiposity categories was BMI ≤25 kg/m^2^

^a^Incident cases/whole sample Diabetes prevailed in 100 (46 M/54 W) participants at baseline

Bold numbers denote significant values

Substituting abdominal obesity for the two BMI categories (Additional file 1: Table S1) replicated the changes in magnitude and sex difference stated for overall adiposity. Whereas only 48 % of the CHD risk was retained by abdominal obesity in men, attenuating the RR to borderline significance, 64 % of the risk was retained in women, rendering persistence of independent significance in RR by abdominal obesity,

Table 3 shows prediction of CHD mortality and incident diabetes by continuous BMI values in each gender, adjusted for SBP, total cholesterol and fasting glucose. For incident diabetes, sex- and age-adjusted BMI was associated with an HR 1.73 per 1 SD increment. When all 3 mediators were added to the model, the HR of BMI was reduced merely as little as less than 3 %: to 2.05 in men and to 1.52 in women.

**Table 3:** Cox regression models for the prediction of sex- and- age-adjusted CHD mortality and incident diabetes by 1SD of body mass index adjusted also for mediators, by gender

	Total		Men		Women	
	HR (95 % CI)	*p*	HR (95 % CI)	*p*	HR (95 % CI)	*p*
CHD mortality	146/2158		86/1015		60/1143	
Body mass index, 5 kg/m^2^	**1.12** (1.10–1.14)	<0.001	**1.11** (1.09–1.13)	0.001	**1.14** (1.11–1.17)	<0,001
Adj. Systolic BP	1.14 (0.95–1.37)	0.16	0.96 (0.72–1.28)	0.81	**1.31** (1.03–1.66)	0,025
Adj. total cholesterol	1.23 (1.05–1.45)	0.010	1.05 (0.81–1.37)	0.70	1.14 (1.11–1.17)	<0,001
Adj. fasting glucose	1.22 (1.03–1.44)	0.021	1.04 (0.80–1.36)	0.76	1.38 (1.12–1.72)	0,003
Adj. SBP & T Chol.	1.11 (0.93–1.34)	0.25	0.94 (0.71–1.26)	0.70	1.27 (0.99–1.62)	0,051
Adj. SBP & FPG	1.12 (0.93–1.35)	0.21	0.93 (0.70–1.24)	0.63	**1.30** (1.03–1.64)	0,025
Adj. T Chol & FPG	1.19 (1.00–1.41)	0.050	1.11 (0.78–1.33)	0.90	1.34 (1.07–1.67)	0,008
Adjusted for all 3 factors	1.10 (0.91–1.32)	0.31	0.91 (0.68–1.22)	0.55	**1.26** (1.00–1.60)	0,050
Incident diabetes	284/2136		146/1014		138/1122	
Body mass index, 5 kg/m^2^	**1.73** (1.54–1.93)	<0.001	**2.11** (1.75–2.54)	0.001	**1.56** (1.35–1.79)	<0,001
Adj. Systolic BP	**1.71** (1.52–1.93)	<0.001	**2.13** (1.73–2.63)	0.001	**1.54** (1.32–1.79)	<0,001
Adj. total cholesterol	1.71 (1.53–1.91)	0.001	2.06 (1.70–2.49)	0.001	1.56 (1.35–1.79	0,001
Adj. fasting glucose	1.71 (1.52–1.91)	0.001	2.10 (1.73–2.55)	0.001	1.55 (1.34–1.78	0,001
Adj. SBP & T Chol.	1.70 (1.50–1.92)	0.001	2.09 (1.69–2.58)	0.001	1.53 (1.32–1.79)	0,001
Adj. SBP & FPG	**1.68** (1.49–1.90)	<0.001	**2.09** (1.69–2.58)	0.001	**1.52** (1.31–1.77)	<0,001
Adj. T Chol & FPG	1.69 (1.51–1.90)	0.001	2.03 (1.67–2.48)	0.001	1.55 (1.34–1.78)	0,001
Adjusted for all 3 factors^a^	**1.67** (1.48–1.89)	<0.001	**2.05** (1.65–2.54)	0.001	**1.52** (1.31–1.77)	<0,001

Female sex was protected against diabetes at HR 0.58 (0.45; 0.75)

1 SD considered here for: systolic BP 25 mmHg, total cholesterol 40 mg/dl, glucose 25 mg/dl

^a^Glucose was the only other significant determinant of diabetes

Numbers in bold denote significant values

CHD mortality was, however, determined only by a significant HR 1.12 by the sex- and age-adjusted BMI. This effect was fully mediated in men by total cholesterol and glycemia and was more than offset by SBP. Women, in contrast, disclosed an inverse mediation effect by the 3 risk factors, so that HR of BMI rose to 1.26 (95 % CI 1.00; 1.60).

Replacing in model 3, Table 3, HDL-C with apoA-I (values were missing in 18 % of the sample) showed tendency to protect against CHD in women (*p* = 0.062) along with mild attenuation of the RR of obesity (*p* = 0.053), while apoA-I was not protective in men (*p* = 0.48), age, SBP and total cholesterol retaining their significant predictive abilities.

## Discussion

This population-based prospective study on middle-aged Turkish adults seeking the direct and mediated effect of adiposity on cardiometabolic risk demonstrated following findings dependent on type of cardiometabolic condition and gender. A) BMI determined the development of type-2 diabetes at a 1.5- to 2-fold HR, more so in men than women, and was virtually *not* mediated by a combination of SBP, total cholesterol and glucose levels. B) Regarding incident CHD, overweight displayed a non-significant tendency to confer risk that was little modified in either gender by the 3 mediators. C) Compared with normal-weight, obesity imparted a significant ~2-fold CHD risk. In men, ¾ of this excess risk was mediated especially by SBP, and by total cholesterol and glucose. In women, in contrast, 60 % of the excess risk was retained by BMI, leaving the remainder to the mediation of the major risk factors. D) BMI, though a significant modest predictor of CHD mortality, was mediated by the conventional risk factors in males, but retained substantially greater independent risk in females. These findings, collectively, suggest that other determinant factors are mediated by BMI/obesity for the risk of diabetes and CHD and, in women, for the risk of CHD by obesity. CHD risk of obesity in men was largely conferred by SBP-total cholesterol, and little by obesity.

Noteworthy is that the overall obesity category was predominated by females whereas the referent “normal-weight” category was so by males (Additional file 2: Figure S1).

Adipose tissue, particularly tissue from visceral-fat deposits, secretes potential mediators in the development of chronic diseases. Obesity is characterized by abnormal adipokine production and the activation of several proinflammatory signaling pathways, resulting in the induction of several biological markers of inflammation [16]. Resistin and tumor necrosis factor TNF-α are implicated in inducing atherogenic adipokines, such as plasminogen activating inhibitor-1 and interleukin-6, and inhibiting adiponectin. TNF-α activates also nuclear factor-(NF-)kB which may mediate hypertension and endothelial dysfunction. In obese patients, macrophage and lymphocyte infiltration in adipose tissue may strongly contribute to obesity-related metabolic dysfunction and chronic inflammation [17].

### Diabetes risk is overwhelmingly determined by BMI and little by glucose

Age-standardized adult diabetes prevalence globally rose since 1980 more rapidly in women than men [18]. The development of diabetes is recognized to be largely co-determined by BMI with which our findings concur. Noteworthy is that added adjustment for fasting glucose hardly attenuated the HR of BMI for diabetes in either or both sexes. This may be related to lipoprotein[Lp}(a)-activated autoimmunity [19] which determines glycemia and concomitantly mediates rise in BMI. In the meta-analysis by Singh et al. [20] RRs for 5 kg/m^2^ higher BMI for ages 55–64 was 2.32 (2.04–2.63) for diabetes and ranged for all adult ages from 3 to 1.4. RRs for the estimated effect of BMI were larger in Western cohorts as compared with Asian cohorts in adults <55 years old. Of interest was that the effect of BMI on incident diabetes in Turkish women was only half that found in men. This may be related to the gender difference existing on Lp(a)-induced autoimmunity [19]. Of further interest was that the mediation of systolic BP and total cholesterol (alike of glucose) was virtually negligible.

### CHD risk of overweight little modified by the three mediators

Elevated peripheral vascular resistance and renal salt retention due to higher sympathetic nervous system activity, angiotensin-aldosterone activity and insulin levels [21] can lead to hypertension in people with adiposity which leads also to dyslipidemia. Moreover, enhanced low-grade inflammation may render insulin resistance and diabetes [22]. We concur with the global meta-analysis [7] that the association between adiposity and CHD is not completely explained by the three mediators in men and underline that it is far from being explained in women.

Overweight imparted modest age-adjusted CHD risk (1.33-fold the “normal” weight); and this was attenuated mainly by systolic BP. The attenuation via BP and serum total cholesterol was 45 % in the meta-analysis of the BMI-CHD Collaborators [6] and was one-half (of the unadjusted RR 1.26) via the three mediating risk factors in the meta-analysis of the Chronic Diseases Collaboration [7]. RR of the age-adjusted CHD risk in the current study was also close to the former meta-analysis.

### The two-fold CHD risk of obesity is mediated by risk factors largely in men, modestly in women

Excess CHD risk, compared with normal weight, showed a steep rise in the obesity category relativeto the overweight one; sex-, age- and smoking-adjusted RRs in combined gender were, namely, 2.24 and 1.40, respectively. In comparison to the meta-analysis on global cohorts [7], this basic excess risk displayed gender difference insofar as the RR was by one-third higher in men, but nearly two-fold in women. Though mediation by the 3 risk factors (with 55 % of the excess risk) was closely similar to that in the global cohorts, gender disparity in the current study was more prominent in the mediation by the 3 risk factors: whereas 70 % of the excess BMI risk was thus mediated in men, 60 % was retained by BMI in women. A gender difference of the BMI-mediated risk was not reported in three meta-analyses [5–7] in which sex-stratified analysis was, however, not reported.

The retained CHD risk by BMI may be due to pathways of systemic inflammation, endothelial dysfunction, and thrombogenic factors. Alike the meta-analysis findings, the largest mediation was found for systolic BP, followed by total cholesterol and least for fasting glucose. Obesity mediation by the individual risk factors was markedly different across sexes inasmuch as glycemia did not mediate any BMI risk in men, in contrast to demonstrating the largest mediation (with 14 %) in women. This distribution of obesity-mediated risk across the sexes as well as the retention of the majority of the obesity risk in women support the notion of autoimmune process activated by obesity being far more common in females than males [19]. It is the autoimmune activation (induced by oxidative stress) and thrombo-embolic events rather than BP or cholesterolemia that lead to fatal and non-fatal CHD.

### BMI modest determinant of CHD mortality

The sex- and age-adjusted continuous BMI variable was a significant modest contributor to HR for CHD mortality; yet this risk was fully abolished by mediation of systolic BP in men, indicating this factor assumed the main determinant of CHD mortality. In contrast, the HR was strengthened to over two-fold in women through mediation of fasting glucose, implicating that impaired fasting glucose (IFG) protected Turkish women against CHD death. This is in agreement with our previous report [23] that IFG status in non-diabetic people without MetS is associated with a less adverse cardiovascular risk profile and reduced future CHD risk, lest modulated by the developed MetS, especially in women in whom serum Lp(a), linked inversely to HOMA, is the likely modulator.

The modest magnitude of HR herein is in line with no effects of overweight on cardiovascular mortality [24], or with overall mortality in a meta-analysis [25]. In contrast, in the Prospective Studies Collaboration [26] 5 units of BMI revealed 40 % excess vascular mortality risk above the optimal BMI 22.5-25 kg/m^2^; below this range, BMI was inversely related to overall mortality.

### Sex-modulated risk of fatal and nonfatal CHD imparted by obesity

Excess risk of age- and smoking-adjusted obesity was larger by one-half in women than men. This is at some variance from the findings of a large pooled analysis on 1.4 million individuals [20] in which effects by age, sex or global region of major metabolic risk factors on cardiovascular disease and diabetes were evaluated. At the age group 55–64 years, the RR was largest for systolic BP with respect to hypertensive HD or stroke, for BMI regarding diabetes or CHD, and at much lower effect size for fasting glucose in regard to CHD or stroke. Proportional effects declined with age, were generally consistent by sex, and differed little by region. However, effects of BMI on diabetes were larger in Western compared with Asian cohorts in younger adults [20].

In regard to BMI-mediated CHD risk of fasting glucose and systolic BP, a clear sex disparity was evident: Women had a significant risk through glucose (by 15 % higher than men), while men had such through systolic BP (by 20 % higher). We have evidence that these features are largely gene-dependent. The TT genotype of the CYP19A1 polymorphism encoding the aromatase enzyme involved in the final step of estrogen synthesis showed, namely, interaction with narrow waist girth for hypertension only in men, independent of age and BMI [27]. In Caucasian US women, an interaction between the risk allele of the FTO rs8050136 polymorphism mediated by BMI and low physical activity yielded increased cardiovascular disease risk [28].

*Implications* relate both to the prevention of CHD and DM. Coronary prevention programs should take into account the role of sex and be modified for sex. While addressing the mediators with measures including antihypertensive and lipid-lowering medication may largely be effective in men, addressing the mediators in women will alleviate only slightly the prevention problem. Major improvement in obesity and related proinflammatory state is required for the CHD prevention. As regards prevention of type-2 diabetes, our findings indicate that the overwhelming portion of the risk can be reduced only by weight loss and improvement in the related oxidative stress (mediated by Lp(a)-activated autoimmunity), regardless of gender.

### Limitations and strength

Our basic regression models did not adjust for certain confounding factors such as socioeconomic state, physical activity grade, or diet; this might have affected the BMI-mediated CHD risk, though not to a great extent. Analysis stratified to sex which substantially generated novel knowledge constitutes a major strength. Despite the fact that, due to lack of an oral glucose tolerance test, some cases of actual diabetes may have been missed, analyzing the BMI-mediated effects also for diabetes and CHD mortality in the same cohort represents further strength.

## Conclusions

Gender, fatal or nonfatal CHD or diabetes modulated in middle-aged Turkish adults the mediation of BMI by 3 traditional risk factors, or the retention of residual excess risk by BMI, respectively. Hardly any mediation was observed with respect to diabetes. Compared with normal-weight, obesity imparted a significant over 2-fold CHD risk. In men, this was extensively mediated while in women, 60 % of the excess CHD risk and virtually the entire risk for CHD death were retained by BMI. These findings can be explained by autoimmune activation occurring in a greater proportion of females, a process mainly induced by oxidative damage to Lp(a) in a setting of excess adiposity.

## Additional files

Additional file 1: Figure S1. Comparative sex distribution of the proportions in the three adiposity categories is shown. Though men prevail in “normal weight” and overweight categories, women predominate in obesity by over two-fold. (DOCX 15 kb) Additional file 2: Table S1. Cox regression models for the prediction of incident CHD by presence of abdominal obesity and three mediators, by gender. (DOCX 21 kb)
