# Supplementary material for: Gender-modulated risk of coronary heart disease, diabetes and coronary mortality among Turks for three major risk factors, and residual adiposity risk
Source: BMC Endocr Disord. 2016 Sep 29;16:54. doi: 10.1186/s12902-016-0134-6 (PMC5041572; doi:10.1186/s12902-016-0134-6)
Supplement: Additional file 1: Figure S1. — Comparative sex distribution of the proportions in the three adiposity categories is shown. Though men prevail in “normal weight” and overweight categories, women predominate in obesity by over two-fold. (DOCX 15 kb) [file 12902_2016_134_MOESM1_ESM.docx]

**Table S1**.Cox regression models for the prediction of incident CHD by presence of abdominal obesity and three mediators, by gender

|  | **Total** | | **Men** | | **Women** | |
| --- | --- | --- | --- | --- | --- | --- |
|  | HR | 95% CI | HR | 95% CI | HR | 95% CI |
| *Model 1* | 405/2158† | | 185/1014† | | 220/1144† | |
| Sex, female | 0.84 | 0.67; 1.60 |  |  |  |  |
| Age, 11 years | **1.75** | 1.59; 1.92 | **1.78** | 1.56; 2.04 | **1.71** | 1.51; 1.94 |
| Current vs. never smoking | **1.56** | 1.21; 2.02 | **1.56** | 1.10; 2.23 | **1.58** | 1.08; 2.31 |
| Former vs. never smoking | 1.26 | 0.96; 1.76 | 1.24 | 0.82; 1.08 | 1.28 | 0.66; 2.51 |
| Abdominal obesity, ≥95/88 cm | **1.77** | 1.42; 2.21 | **1.67** | 1.23; 2.26 | **1.94** | 1.39; 2.71 |
| *Model 2* | 405/2158† | | 185/1014† | | 220/1144† | |
| Sex, female | 0.96 | 0.82; 1.32 |  |  |  |  |
| Age, 11 years | **1.54** | 1.38; 1.71 | **1.56** | 1.35; 1.82 | **1.49** | 1.30; 1.73 |
| Current vs. never smoking | **1.68** | 1.29; 2.17 | **1.67** | 1.17; 2.40 | **1.63** | 1.11; 2.40 |
| Former vs. never smoking | 1.25 | 0.89; 1.75 | 1.19 | 0.78; 1.80 | 1.34 | 0.68; 2.63 |
| Abdominal obesity, ≥95/88 cm | **1.48** | 1.14; 1.80 | *1.32* | 0.96; 1.81 | **1.60** | 1.14; 2.24 |
| Total cholesterol, 40 mg/dl | **1.21** | 1.09; 1.35 | **1.21** | 1.03; 1.41 | **1.22** | 1.06; 1.41 |
| Fasting glucose, 25 mg/dl | **1.12** | 1.04; 1.21 | 1.04 | 0.92; 1.18 | **1.19** | 1.08; 1.30 |
| Systolic BP, 25 mmHg | **1.39** | 1.26; 1.53 | **1.53** | 1.29; 1.81 | **1.34** | 1.19; 1.50 |

Abdominal obesity prevailed in 49.3% in men, in 60.8% in women.

†Incident cases/whole sample Diabetes prevailed in 100 (46M/54 W) participants at baseline.

Referent in adiposity category was WC <95/<88 cm
