# Supplementary material for: Gender-modulated risk of coronary heart disease, diabetes and coronary mortality among Turks for three major risk factors, and residual adiposity risk
Source: BMC Endocr Disord. 2016 Sep 29;16:54. doi: 10.1186/s12902-016-0134-6 (PMC5041572; doi:10.1186/s12902-016-0134-6)
Supplement: Additional file 2: Table S1. — Cox regression models for the prediction of incident CHD by presence of abdominal obesity and three mediators, by gender. (DOCX 21 kb) [file 12902_2016_134_MOESM2_ESM.docx]

**Figure S1** Comparative sex distribution of the proportions in the three adiposity categories is shown. Though men prevail in “normal weight” and overweight categories, women predominate in obesity by over two-fold.
